# Experiences, Beliefs, and Values Influencing Midwives’ Attitudes Toward the Use of Childbirth Interventions

**DOI:** 10.1111/jmwh.13392

**Published:** 2022-08-02

**Authors:** Lianne D. C. Zondag, Veronique Y. F. Maas, Antje Beuckens, Marianne J. Nieuwenhuijze

**Affiliations:** ^1^ Care and Public Health Research Institute (CAPHRI) Maastricht University Maastricht the Netherlands; ^2^ Department of Obstetrics and Gynaecology Erasmus MC Rotterdam the Netherlands; ^3^ Haarlem the Netherlands; ^4^ Research Centre for Midwifery Science Zuyd University Maastricht the Netherlands

## Abstract

**Introduction:**

Intervention rates in perinatal care vary between and within countries, without populations’ characteristics as a full explanation. Research suggests that one factor in this variation might be the attitudes of perinatal health care providers. Systematic knowledge on the background of midwives’ attitudes and how this influences the use of interventions is limited. The study aim was to to explore experiences, beliefs, and values that influence midwives’ attitudes toward interventions in perinatal care.

**Methods:**

A qualitative study using in‐depth interviews with primary care midwives (n = 20) in the Netherlands. The interviews were performed in June 2019 and combined a narrative approach with a semistructured interview guide. Inductive content analysis was applied.

**Results:**

We identified 2 main themes: attitudes toward interventions and influences on midwives’ attitudes. The midwives in our study described their attitudes toward interventions as oriented to either wait and see or check and control. Care based on wait and see displayed a more supportive style of behavior, and care based on check and control appeared to display a more directive style of behavior. In the theme of influences on midwives’ attitudes, 3 subthemes emerged: experiences in collaboration, trust and fear, and woman‐centeredness.

**Discussion:**

Midwives with a wait and see attitude seem to have a more restricted approach toward interventions compared with midwives with a check and control attitude. Midwives need to be aware how their experiences, beliefs, and values shape their attitudes toward use of interventions. This awareness could be a first step toward the reduction of unwarranted interventions.

## INTRODUCTION

The rates of childbirth interventions have shown a steady increase in the last decades.^1^ For example, in 2000 a caesarean birth was performed in 12.1% of all births worldwide. This number almost doubled in 2015, when the caesarean birth rate was 21.1%.^2^ Interventions during pregnancy, childbirth, and the postpartum period can prevent both perinatal morbidity and mortality. Inadequate access to timely childbirth interventions jeopardizes positive birth outcomes.^3^ Conversely, medicalization of normal antenatal, natal, and postnatal care by using unnecessary interventions has negative consequences and seems an even larger problem worldwide.^3^, ^4^ Childbirth interventions have the potential to harm women, physically and mentally, and their newborns, and therefore unnecessary use should be avoided.^5^, ^6^ In addition, overuse of childbirth interventions leads to higher health costs.^7^

Worldwide, the Netherlands is known for its low intervention rates and high rates of home births. In this country, childbirth has long been defined as a physiologic process where the woman can choose to give birth at home or in a hospital under the care of a midwife.^8^ Dutch midwives are supposed to be the guardians of physiology and advocate for nonintervention in physiologic childbirth.^9^ Two‐thirds of the midwives work in community group practices with 2 to 5 midwives as independent health professionals paid by insurance companies. They provide antenatal, natal, and postnatal care to healthy women registered with the practice, who can choose to give birth with their midwife at home or in the hospital.^10^ Dutch primary care midwives are an integrated part of the Dutch perinatal care system and collaborate with one or more hospitals in their region.^10^ The other third of Dutch midwives are employed by hospitals and take care of women with complicated pregnancies or births under the supervision of obstetricians. Almost 90% of pregnant women start antenatal care in midwife‐led primary care.^11^ Midwives refer women to obstetrician‐led care when risks of adverse outcomes increase or complications arise.^12^ Criteria for referral are described in the obstetric indication list of 2003; however, every primary care midwife has the autonomy to refer to obstetrician‐led care at any point in care. In addition, primary care midwives can make autonomous decisions together with the woman to perform certain childbirth interventions such as artificial rupture of membranes, episiotomy, and postpartum administration of oxytocin.

During the past decades, a rise in childbirth interventions has been described in the Netherlands.^13^ Additionally, large variations in childbirth interventions are described between regions in the Netherlands and between Dutch primary midwifery practices, without differences in neonatal or maternal outcomes.^12^, ^13^, ^14^ These variations cannot be explained by variation in populations’ characteristics, and it remains unclear why a group of autonomous midwives in the same small country shows such a wide variation in use of childbirth interventions.^12^, ^13^ Previous research showed a positive correlation between intervention rates in midwife‐led care and obstetrician‐led care within the same region.^15^ This suggests that culture in the work environment influences care providers within the same region toward comparable use of interventions and that variations are not merely individual. The wide variation of referral rates among Dutch midwives supports the theory that attitudes toward use of interventions differ between midwives.^13^ Furthermore, fundamental differences between midwives in their willingness to support the physiology of pregnancy and childbirth are known to result in different individual attitudes toward childbirth interventions.^16^

**QUICK POINTS**

✦ Two attitudes toward use of interventions were identified: an attitude oriented to check and control and an attitude oriented to wait and see.
✦ Care based on wait and see displayed a more supportive style of behavior.
✦ Care based on check and control displayed a more directive style of behavior.
✦ Midwives with a wait and see attitude tended to use fewer interventions in care.

According to the theory of planned behavior, individual experiences, beliefs, and values influence a person's attitude, which subsequently, together with social influences and self‐efficacy, shapes the intention to perform a specific behavior.^17^, ^18^ Therefore, the midwife's attitude is an interacting factor in the use of interventions. However, systematic studies on the background of midwives’ attitudes and how this influences the use of interventions in perinatal care are limited. The purpose of this study was to explore deeper and to identify the experiences, beliefs, and values that shape midwives’ attitudes and how these factors influence their clinical decision‐making when deciding to use interventions. These findings can direct further research into deeper understanding of the factors that influence practice variation in the use of interventions.

## METHODS

### Study Design

We conducted a qualitative study using in‐depth interviews that combined a narrative approach with a semistructured question route to indicate relevant topics (Table 1). In accordance with the narrative approach, we invited the participating midwives to elaborate and share stories about situations during pregnancy, birth, and the postpartum period where decisions were made on the use of interventions. At start of the interview, midwives described their definition of a childbirth intervention. The narrative approach made it possible to explore which experiences, beliefs, and values of the midwives were important during their clinical decision‐making.^19^ Using a semistructured interview helped to ensure that all relevant topics were explored. Although acknowledging that not all individuals in perinatal care refer to themselves as women, we have chosen to use *women* and feminine pronouns.

**Table 1:** Questions for the semi‐structured interview guide

**Questions**
What do you think is the definition of a medical intervention in midwifery care?
Please describe a situation during maternity care (pregnancy, birth, or postpartum) in which many interventions were performed.
Please describe a situation during maternity care (pregnancy, birth, or postpartum) in which few interventions were performed.
When do you experience the need to perform a medical intervention?
Which factors influence whether or not a medical intervention is performed?
How do you involve clients in the performance of interventions?
How do you evaluate the use of interventions in midwifery care?

### Setting and Participants

Based on data from the Dutch national register Perined, we selected midwifery practices varying in rates of home birth, episiotomy, and referral during pregnancy or birth. In total, we invited midwives from 56 practices for an interview. Twenty‐two primary care midwives accepted the invitation, and we interviewed 20 midwives in June 2019. We excluded one midwife because a colleague from the same practice was already interviewed and one midwife because no suitable date could be found. The reason for the nonresponse of the 34 remaining practices is unknown.

### Data Collection

Before the interviews, we distributed a short questionnaire to collect the participants’ demographic characteristics and usage of childbirth interventions in their midwifery practice. Interviews were face‐to‐face conversations at a safe setting chosen by the participant. The interviews were conducted by 4 interviewers who had a midwifery background but no personal relationships with the participants. We audio‐recorded and transcribed the interviews verbatim for analysis. The transcribed interviews were sent to the participants for participant validation; however, no adjustments were requested. The anonymized and encrypted transcripts, together with field notes, were stored safely and locked, accessible solely to the research team.

### Rigor and Reflectivity

We used several strategies to ensure methodological rigor.^20^ The first 2 interviews of each interviewer were observed by a colleague interviewer to ensure consistency and quality. After the first 4 interviews, the research team made small adjustments to the interview guide to strengthen the narrative approach through peer debriefing. Field notes were kept from each interview to achieve data triangulation. Throughout the study, we reflected on the analytic process as a group to arrive at consensus through investigator triangulation. We used the Standards for Reporting Qualitative Research (SRQR) as guidance for writing the current article.^21^ The SRQR reporting checklist is provided in Supporting Information: Appendix S1.

### Ethical Considerations

According to the Medical Research Involving Human Subjects Act (WMO) in the Netherlands, formal ethical approval by a research ethics committee is only required for medical research where participants are subject to interventions or procedures or are required to follow specific, research‐related rules of behavior.^22^ None of these apply to this research. All the midwives gave informed consent and were aware of their rights.

### Data Analysis

Subsequently, we performed an inductive content analysis^23^, ^24^ using the online software program Dedoose (version 8.3.17). The first and last author developed a preliminary coding scheme based on the data of 3 randomly chosen interviews and the structure of the attitude–social influence–self‐efficacy model.^17^, ^18^ The final coding scheme emerged during further analysis based on consensus. We grouped the codes into subthemes and themes by examining the commonalities, differences, and relationships within and among the interviews. After 11 interviews, we reached saturation on the level of themes and subthemes, but we analyzed 2 additional interviews for confirmation. We read the remaining 7 interviews to check whether any codes or themes had been missed and confirmed the stated themes. During the analysis, a theory emerged on midwifery styles of behavior toward interventions and was supported by the themes and subthemes.^23^

## RESULTS

All participating midwives worked in primary care in the Netherlands, and they varied in age, place of education, years of experience, and midwifery practice characteristics (see Table 2). Five midwives were educated in Belgium, Switzerland, or the United Kingdom, reflecting the overall education background of midwives in the Netherlands.^25^

**Table 2:** Characteristics of Participants (n = 20)

**Characteristic**	**Value**
**Age, mean (range), y**	42.8 (24‐60)
**Work experience, mean (range), y**	16.4 (2‐30)
**Place of education, n**	
The Netherlands – North	4
The Netherlands – South	4
The Netherlands – Amsterdam	3
The Netherlands – Rotterdam	4
Abroad (Belgium, United Kingdom, Switzerland)	5
**Size of midwifery practice, n** **^a^**	
Small	6
Medium	9
Large	5
**Location, n**	
North	5
East	4
Central	4
West	3
South	4

^a^ Size of the midwifery practice is based on the number of annually completed cases. The definition of a case is provision of complete care during pregnancy, labor, and postpartum. Size is divided into small (<80 completed cases), medium (80‐300 completed cases), and large (>300 completed cases).

The following 2 main themes were evident in the data: attitudes toward interventions and influences on midwives’ attitudes. Within the theme of influences on midwives’ attitudes, we found 3 subthemes: experiences in collaboration, trust and fear, and woman‐centeredness. Finally, an emerging theory on midwifery styles of behavior toward childbirth interventions is presented.

### Attitudes Toward Interventions

All midwives in our study agreed that pregnancy and childbirth are physiologic processes. Midwives told us about supervising the progress of labor and monitoring the condition of the woman, partner, and child as being their specific responsibility. Some midwives in our study did not only define medical procedures as interventions but also included psychosocial aspects like being present and various forms of coaching as part of their definition of an intervention:

“Everything [that] influences the natural behavior of the woman giving birth … In case a woman needs some encouragement, such as ‘you can do this, it's going well.’ This is also an intervention for me.” (Midwife 12)

Talking about interventions, some midwives in our study seemed to have a more wait and see approach toward the care provided in pregnancy, childbirth, and the postpartum period. These midwives expressed that they preferred to be present in the background, meaning to be somewhere in the house or in the corner of the room and only interfering when invited by the woman and her partner,

“… which is a birth where the health care providers are invisible, where a woman can determine her own posture, determine her own coping strategy and have as little interference as possible.” (Midwife 12)

Midwives with a more wait and see approach reported that they used guidelines as a tool in clinical decision‐making. Other midwives in our study explained they considered it important to follow the national guidelines or local protocols in all or nearly all circumstances. These midwives seemed to have a more check and control approach. They felt that by strictly following the national guidelines or local protocols, the best possible physical outcomes could be guaranteed. These midwives emphasized that interventions helped them to obtain additional information about possible pathology, which gave them a feeling of certainty and safety. In the following quotation, an example of the check and control approach is given. However, it should be noted that an oral glucose tolerance test (OGTT) is not standard care for all women in the Netherlands; it is only indicated and offered when women have specific risk factors.

I agree that if someone is really overweight or has diabetes in the family, it's good to perform an OGTT. It gives me the feeling of “I have checked it, so that is good” … That also provides a bit of control, a bit of certainty. (Midwife 17)

The differences in attitude toward the use of interventions between midwives with a wait and see approach and midwives with a check and control approach became most clear when midwives talked about prevention of pathology, indications for ultrasound scans, and their belief that they need to advocate for nonintervention in physiologic childbirth. In their stories, the midwives expressed a variation in the application of interventions like artificially rupture of membranes, vaginal examinations, or postpartum administration of oxytocin.

### Influences on Midwives’ Attitudes

When describing the background of their attitudes toward interventions, the midwives in our study mentioned different experiences, beliefs, and values, such as experiences in collaboration, trust and fear, and woman‐centeredness.

#### Experiences in Collaboration

In all interviews, midwives discussed how collaboration with other health care providers influenced their attitudes toward the use of interventions. Regarding collaboration with providers of obstetrician‐led care, midwives with a check and control approach repeatedly mentioned that they wanted to satisfy the obstetrician and seemed to be influenced by the perceived hierarchy. These midwives reported that they performed more interventions then recommended in the national standard because of this feeling of authority:

“I sometimes think: ‘what does the obstetrician think about it [care management]?’. That is on my mind when I don't achieve a certain result within a certain time.” (Midwife 7)

Other midwives experienced local protocols as restrictive and they often felt obligated to follow them, as this was mutually agreed upon at a local level. They felt internal conflict between conforming to the local agreements and their view on how much value the intervention added. Midwives in our study did not mention any internal conflict about the national guidelines, only about the local protocols:

“The standard offering of OGTT for risk factors is something I feel compelled to do because those are the agreements. But, I'd rather not do it because I don't support this regional management. I feel compelled from the outside …” (Midwife 9)

In our study, most midwives strove for collaboration based on equality with obstetric care providers. Nevertheless, midwives reported experiencing a difficult relationship with obstetricians and hospital‐based midwives because of different perceptions over what constituted the minimum necessary care for women at low risk. Midwives described how they succeeded in defending their physiologic statements in regional collaboration sessions. As a result, the regional protocol included fewer mandatory interventions than had been requested by obstetrician‐led care providers.

“In our region, the postpartum hemorrhage protocol stagnated for a long time. In the end, we managed to cancel the obligation to administer oxytocin by default. So yes, we fight … it feels now and then a bit like fighting, but that ultimately helps to ensure that you don't have to justify yourself for everything every time.” (Midwife 10)

#### Trust and Fear

Trust and fear seemed to be important factors in directing midwives’ attitudes toward the use of interventions. Midwives in our study mentioned that their experiences influenced their fear of complications. They described how experiencing uncomplicated situations boosted their trust in physiologic childbirth and made them feel more restrained toward the use of interventions.

I certainly feared upright births in my early years. After the baby was born, the blood clattered down and I wanted to administer oxytocin right away. After a while, you get used to the noise and you can estimate the blood loss better, and you hold off administering oxytocin. (Midwife 14)

However, more often, midwives talked about the emotions they felt after a perinatal emergency such as a postpartum hemorrhage or fetal distress. They described feeling helpless and afraid when these situations happened and feeling uncertain and unsafe during subsequent births. The feelings described were mostly related to childbirth and rarely to a situation occurring in pregnancy, such as fetal growth restriction: “After that case [neonate with asphyxia], when I noticed a little dip in the fetal heartbeat, I immediately thought ‘oh no, not again.’” (Midwife 3)

It appeared that midwives could react in 2 ways to these stressful experiences. One group became more defensive, felt they needed to do more interventions, and performed more interventions than they had in previous pregnancies or births. This group practiced the check and control approach to feel more certain and safe. The other group of midwives reflected on having feelings of uncertainty and fear that a perinatal emergency or complication would reoccur. They suggested that they needed to regain confidence but were aware of this response and the impact it might have on their use of interventions. As described in the following quotation, these midwives consciously did perform more interventions in later situations:

Afterward, all midwives in my practice wanted to check blood when a woman had a little itching [after a fetal death caused by cholestasis]. But it was just one case, and we cannot implement a new treatment based on one case. (Midwife 11)

Besides fear of complications or perinatal mortality or morbidity, midwives spoke about their fear of a legal complaint or claim.

#### Woman‐Centeredness

Most midwives in our study talked about woman‐centered care in relation to their attitudes toward the use of interventions. They talked about how woman‐centered care was part of their daily practice and about the difficulties they encountered in providing this care. They highly valued the wishes of the woman and strove to meet these wishes as best as possible. When the woman had other preferences than her midwife's concerning perinatal care management, midwives with a more wait and see approach told us they would support the woman in her choice. Other midwives expressed difficulties supporting the woman's care wishes when these wishes differed from their own preferred management. They described a dilemma between the values of woman‐centeredness and safety. For example, in a critical situation, they did not ask for informed consent before they acted because obtaining informed consent felt like losing time.

We explain [in the pregnancy]: “… if you have a lot of blood loss we will administer oxytocin.” They will agree to it. During birth, you also need to obtain informed consent. I find that difficult. Sometimes I just want to administer it. (Midwife 15)

Midwives who told us about their difficulties with supporting a woman's preference when it differed from the national guidelines and local protocols were more inclined to persuade the woman to follow the midwife's health care management plan. This often included more interventions. Midwives gave examples of situations in which they persuaded or overruled the woman. In their stories, the midwife appeared to be the main subject instead of the woman.

I look at what the patient wants. If I find it medically responsible, they can do anything they want to. But if things are not going in the right direction, I will put a stop to it. I try to explain why, and usually, they listen. (Midwife 20)

All midwives mentioned that workload sometimes influenced the extent to which they provided woman‐centered care and influenced their use of a particular intervention. Notably, midwives with a more wait and see approach described changing their work circumstances to be able to provide care from this approach. They choose to work in smaller teams or in shorter shifts, or even stopped working in a group practice and started working in caseload midwifery to create more time for each woman and experienced a lower workload. Midwives with a more check and control approach talked about the Dutch perinatal care system and the consequences of this system for the workload of a primary care midwife as an entrepreneur.

### Supportive and Directive Styles

**Figure 1:**
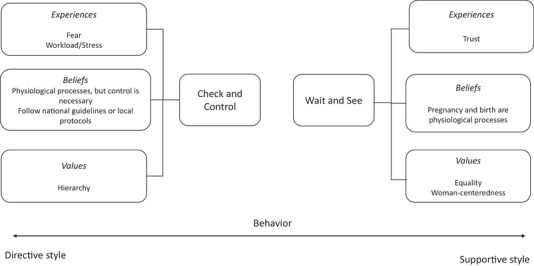
Experiences, Beliefs, and Values Underpinning Attitudes and Behavior Toward Use of Interventions

From the analysis, 2 attitudes emerged that influenced midwives’ approach toward perinatal care: a wait and see attitude and a check and control attitude. In the 3 subthemes, the midwives in our study described what experiences, beliefs, and values influenced their attitudes. We summarize this in Figure 1.

Midwives with a wait and see attitude described a more restricted approach toward interventions than midwives with a more check and control attitude. They described childbirth as a physiologic process that only needs an intervention when pathology occurs or at a woman's request. Experiences of uncomplicated births reinforced trust in the physiologic childbirth. Midwives with this attitude emphasized the importance of collaborating with health care providers in obstetrician‐led care on an equal basis. It seemed that care based on a wait and see attitude resulted in a more supportive style of behavior.

Midwives with a check and control attitude toward perinatal care tended to use more interventions compared with midwives with a more wait and see attitude. These midwives also described pregnancy, childbirth, and the postpartum period as physiologic processes. However, they relied on national guidelines and local protocols for reassurance about the process and indications for performing interventions. These midwives seemed to be more affected by authority in the collaboration with obstetric care providers and the interaction with the woman. Care based on a check and control attitude seemed to result in a more directive style of behavior.

The 2 attitudes that emerged toward the use of interventions do not translate into a definitive distinction of each midwife's individual style of behavior. In our analysis, participants showed a tendency toward a stronger orientation to the wait and see attitude combined with a more supportive style of behavior or to the check and control attitude combined with a more directive style. Still, certain circumstances could prompt midwives to use the other style of behavior.

## DISCUSSION

Midwives’ attitudes toward interventions presented as an attitude oriented to wait and see and an attitude oriented to check and control. Coping with experiences of complications or complaints, as well as values of collaboration with women or health care providers in obstetrician‐led care, influenced midwives’ attitudes. It appears that midwives with a wait and see attitude have a more restricted approach to interventions compared with midwives with a check and control attitude. The care of midwives based on a wait and see attitude suggested a more supportive style of behavior, whereas care based on a check and control attitude suggested a more directive style of behavior.

### Two Paradigms

The wait and see and check and control attitudes match the sociological framework of the social and medical model of care.^26^ In practice, a whole range of combinations of the 2 ways of operating can be seen.^26^ In accordance with the social model, midwives with a wait and see attitude focus on normality of childbirth, social support, and an active involvement of the individual woman. In accordance with the paradigm of trust,^27^ these midwives build a relationship with the woman through open communication and equality. The midwife–woman relationship is the vehicle through which trust is built and personalized care is provided, and it contributes to a woman's feeling of empowerment.

In contrast, the medical model of care focuses on risk and means of risk reduction; in this model, women are less involved in their care.^26^ Midwives with a check and control attitude also believe that normal childbirth requires medical control to guarantee safety and apply interventions at the earliest sign of pathology, as in the medical model. This attitude is more influenced by the paradigm of risk.^28^ In the last century, midwifery care has become more technologically oriented, and the number of hospital births has grown, creating an increasing reliance on standardized procedures and a prominent place for risk management.^26^, ^29^, ^30^ As such, the standardization of care by guidelines and audits of health care provision have brought about a shift in focus away from the trust in the normality of childbirth and professional autonomy.^30^, ^31^

Perinatal care in the United States is more influenced by the paradigm of risk, resulting in a care system that more strongly treats birth as an event with a high potential of pathology.^32^ Most midwives work in a hospital setting, where the autonomy of a midwife differs greatly between hospitals. In the Netherlands, primary care midwives work in collaboration with hospitals but can make autonomous decisions about certain nationally regulated childbirth interventions, for example, external version of a breech.^10^, ^14^, ^15^ On the other hand, certain interventions, such as epidural analgesia, are only accessible in obstetric‐led care, and women are referred if wanting or needing them.^10^, ^15^ Still, our study shows that attitude toward childbirth interventions is influenced by various factors even though the midwife has the autonomy to make her own decisions together with the woman. These findings can make midwives from both countries aware of the significant influence of their attitudes on the way they practice midwifery. Influencing factors on attitude did not depend on the work setting of the midwife, so the results of this study probably also apply to midwives working in a hospital.

### Changes in Dutch Midwifery Care

According to social science, different responses to risk depend on the perception of an individual or group.^33^ Childbirth in the Netherlands has long been defined as a physiologic process under the care of a midwife.^8^ Education programs in the Netherlands still teach student midwives the basic attitude that pregnancy and childbirth are physiologic processes needing minimal interventions.^34^ However, studies on referral rates among Dutch midwives show a movement toward medicalization of midwifery care in the Netherlands.^14^ It appears that some midwives can retain their basic physiologic attitude better than their colleagues. For example, this study provided insight into different coping mechanisms of midwives according to fear of complications, resulting in variation in application of interventions.

In recent years, there has been a call in the Netherlands to provide more woman‐centered care, and the term *watchful attendance* has been introduced.^35^ Watchful attendance is a combination of continuous support, clinical assessment, and responsiveness.^35^ Midwives perform regular clinical checks integrated into the whole dynamic of care. Many benefits of care with watchful attendance have been described;^36^ however, midwives’ motivations and individual skills greatly influence whether and how this care is provided.^35^, ^37^ In addition, discussion continues that the current official workload is too high to effectuate woman‐centered care in all its aspects.^38^ The number of midwives per 100,000 women in the Netherlands is only 31.1, making it one of the 5 Western countries with the lowest number of midwives.^39^ In our study, midwives who wanted to provide woman‐centered care made changes in their practice toward working in smaller teams or started working as a caseload midwife, expecting a reduction in salary.

### Limitations and Strengths

Our sample of 20 midwives resulted in a wide variety of participants and information about experiences, beliefs, and values. This study included only midwives working in primary care, so its results cannot be generalized to those working in the hospital setting. Several steps were taken to minimize bias, such as using an interview guide, member checks, and interviews of midwives by people unknown to them. The researchers were aware they had preconceived ideas, knowledge, and understanding that possibly influenced the execution and outcomes of the study. To ensure that interpretations were valid and grounded in reality, the researchers engaged in continual self‐reflection, and the first, second, and last author collaborated in the analysis. Further research should give insight into which knowledge, skills, barriers, and supports interact with intentions to perform a certain behavior, to convert an intention to actual behavior.

### Recommendations for Practice

For appropriate use of interventions during pregnancy, birth, and the postpartum period midwives should learn to balance between the 2 attitudes and corresponding styles of care. Awareness of their underlying personal experiences, beliefs, and values can provide midwives insight into their attitudes toward interventions. If they feel supported by a broader movement toward watchful attendance, this could be a first step toward behavioral change and the reduction of unwarranted interventions.

## CONCLUSION

All midwives in our study had the intention to only perform interventions when appropriate. It seems that midwives with a more wait and see attitude have a more restricted approach toward interventions compared with midwives with a more check and control attitude.

## CONFLICT OF INTEREST

The authors have no conflicts of interest to disclose.

## Supporting information


**Appendix S1**. SRQR Reporting Checklist
